# Mincle, an Innate Immune Receptor, Is Expressed in Urothelial Cancer Cells of Papillomavirus-Associated Urothelial Tumors of Cattle

**DOI:** 10.1371/journal.pone.0141624

**Published:** 2015-10-29

**Authors:** Sante Roperto, Valeria Russo, Iolanda Esposito, Dora Maria Ceccarelli, Orlando Paciello, Luigi Avallone, Rosanna Capparelli, Franco Roperto

**Affiliations:** 1 Dipartimento di Medicina Veterinaria e Produzioni Animali, Settore Malattie Infettive, Università di Napoli Federico II, Napoli, Italia; 2 Dipartimento di Medicina Veterinaria e Produzioni Animali, Settore Patologia Generale, Università di Napoli Federico II, Napoli, Italia; 3 Dipartimento di Medicina Veterinaria e Produzioni Animali, Settore Fisiologia, Università di Napoli Federico II, Napoli, Italia; 4 Dipartimento di Agraria, Università di Napoli Federico II, Napoli, Italia; 5 Dipartimento di Biologia, Università di Napoli Federico II, Napoli, Italia; Duke University Medical Center, UNITED STATES

## Abstract

**Background:**

Mincle, macrophage-inducible C-type lectin, is a member of C-type lectin receptors. It plays an important role in anti-mycobacterial and anti-fungal immunity. Furthermore it senses dead cells through its primary ligand SAP130.

**Materials and Findings:**

We examined ten urothelial tumors of the urinary bladder of cattle. Eight of them expressed E5 cDNA of bovine papillomaviruses type 2 (BPV-2) and type 13 (BPV-13) that belong to Deltapapillomavirus genus. Two of them were not examined for detection of E5 cDNA. Mincle expression appeared to occur in urothelial neoplastic cells only. No mincle expression was detected in urothelial cells from healthy cattle. Mincle expression was characterized by a membranous pattern in papillary urothelial cancers; isolated and/or clustered urothelial cells showing a strong cytoplasmic immunoreactivity were primarily seen in invasive urothelial cancers.

**Conclusion:**

This is the first study about the expression of mincle in veterinary oncology and the first report which describes the expression of functional mincle receptor in neoplastic cells in medical literature. As it has been shown that urothelial cancer cells have the ability to function as antigen-presenting cells (APCs), it is conceivable that mincle expression is involved in the presentation of cancer cell antigens to cells of the immune system. Furthermore, since expression of mincle contributes to the control of *Mycobacterium bovis* BCG infection, this study has exciting clinical implications in comparative medicine keeping in mind that Bacillus Calmette-Guérin (BCG) immunotherapy is currently the most effective treatment of non-muscle invasive bladder cancer in man. Mincle expression in urothelial tumor cells warrants further study to better understand the role, if any, of this receptor in bladder cancer. Future studies will provide insights in the role of mincle receptor of urothelial cancer cells in antitumor immunotherapy.

## Introduction

C-type lectin receptors (CLRs) comprise a large superfamily of proteins primarily expressed on myeloid cells where they effectively function as pattern recognition receptors (PRRs) [1]. CRLs include more than a thousand identified members from several animal species [2] and are believed to be the fourth family of pattern-recognition receptors [3]. CLRs recognize a diverse range of ligands from ‘nonself’ (pathogen-associated molecular patterns—PAMPs), ‘damaged self’ (damage-associated molecular patterns—DAMPs) or ‘altered self’ (tumor-associated molecular patterns—TAMPs) [1].

Mincle, macrophage-inducible C-type lectin (also called as CLEC4E), is a member of CLRs. It was originally identified as a transcriptional target of NF-interleukin-6 (IL-6) mainly expressed on professional antigen-presenting cells (APCs), such as macrophages, dendritic cells (DCs), B cells, and neutrophils [4]. The expression level of mincle in the steady-state condition is very low; however, it is strongly upregulated after exposure to different inflammatory signals [5]. Mincle appears to be selectively associated with the Fc gamma receptor (FcγR) and activates macrophages to produce inflammatory cytokines and chemokines [6]. Mincle is a key receptor for the mycobacterial cord factor trehalose dimycolate (TDM) thus being an important modulator of the antimicrobial immunity [7,8]. Mincle has a crucial role in antifungal immunity as it recognizes some pathogenic fungi such as *Candida albicans*, *Malassezia* species and *Fonsecaea pedrosoi*, the causative agent of chromoblastomycosis [9–11]. Furthermore, mincle senses dead cells through its primary ligand SAP130 (spliceosome-associated protein 130), a small ribonucleoprotein released from necrotic cells only [6]. Recently, it has been shown that the natural product brartemicin, an inhibitor of cancer cell invasion, is a high-affinity ligand of carbohydrate-recognition domain (CRD) of mincle receptor [12].

Mincle was recently found to be expressed in non-immune cells such as neurons and endothelial cells after ischemic stroke in both mice and humans; therefore, it has been suggested that mincle and its ligand SAP130 participate in the pathogenesis of cerebral ischemia by initiating inflammation [13, 14].

Urinary bladder tumors are very rare in cattle accounting for 0.01% of all bovine malignancies [15]. On the contrary, bladder neoplasms are common in adult cattle that have grazed on pasturelands rich in bracken fern (*Pteridium* spp.) [16–19]. Ptaquiloside, a bracken sesquiterpenoid, and infectious agents are believed to be responsible for bladder cell transformation. Although many bacteria have been found in the urine microbiota of cattle affected by bladder tumors [20], bovine papillomaviruses (BPVs) of Delta genus appear to be the most important infectious agents involved in the etio-pathogenetic mechanisms of bladder carcinogenesis. In particular, bovine papillomavirus type 2 (BPV-2) and type 13 (BPV-13) appear to be the most prevailing virus responsible for bladder tumor in cattle [17, 21–26].

The aim of the present paper is to report the expression of mincle receptor in urothelial cancer cells of naturally occurring papillomavirus-associated urothelial tumors in cattle.

## Material and Methods

### Ethics statement

In this study we did not perform any animal experiments. We collected the samples directly from private and public slaughterhouses; the animals were slaughtered following a mandatory clinical ante-mortem examination as required by European Union (EU) legislation.

### Tumor Samples

Ten bovine urothelial tumor samples and five bladder samples from apparently healthy cows were collected with the permission of the medical authorities in the slaughterhouses named “Barbara Rocco sas” of Simbario (Calabria Region), “Macello Comunale” of Muro Lucano (Basilicata Region), “Cestari Carni srl” of Montesano sulla Marcellana (Campania Region), “Frigo Sud srl” of Nocera Superiore (Campania Region), “Real Beef srl” of Flumeri (Campania Region).

To prevent possible cross-contaminations, both neoplastic and normal bladder samples were immediately divided into several parts. Some parts were frozen in liquid nitrogen and stored at -80°C for subsequent molecular biological analysis. The remaining parts were fixed in 10% buffered formalin for microscopic investigations.

### Histopathology

The tissues fixed in 10% buffered formalin were routinely embedded in paraffin wax. Histologic diagnosis was assessed on 5-μm-thick haematoxylin-eosin (HE)–stained sections using morphologic criteria suggested in the report on the new histological classification of urothelial tumors of the urinary bladder of cattle [19].

### RNA extraction

Total RNA was extracted from urinary bladders of cows using the RNeasy Mini Kit (Qiagen, Milan, Italy), according to the manufacturer’s instructions. The quantity and quality of RNA were assessed using the NanoVue Plus and electrophoresis on 1% agarose gel. Genomic DNA was removed from RNA preparations using RNase-free DNase I Fermentas Life Sciences (Dasit, Milan, Italy).

### Reverse Transcription (RT)-PCR for BPV-2, BPV-13 E5, Mincle, FcγR and CXCL2

Total RNA was transcribed using the iScript cDNA Synthesis Kit (Bio-Rad Laboratories, Milan, Italy) and the reaction was incubated at 25°C for 5 min, 42°C for 30 min, 85°C for 5 min, and then kept at 4°C for 5 min. The synthesized cDNA was analyzed by PCR with specific primers for the BPV-2 E5 ORF (forward, 5’-CACTGCCATTTGTTTTTTTC-3’; reverse, 5’-GGAGCACTCAAAATGATCCC-3’), for the BPV-13 E5 ORF (forward, 5’- CACTGCCATTTGGTGTTCTT—3’; reverse 5’- AGCAGTCAAAATGATCCCAA-3’), for Mincle (forward primer, 5’- GACTGAGGGTCAGTGGCAAT -3’; reverse primer, 5’- GTCCCTTATGGTGGCACAGT-3’), for FcγR (forward, 5’- TTTGGTTGAACAAGCAGCGG—3’; reverse 5’- TGCAACTTGAGTCGGCAGTA -3’) and for CXCL2 (forward, 5’- GCCGCTCCCATGGTTAAGAA—3’; reverse 5’- TCTGTAGGGGCAGGGTCTAC -3’). To evaluate the adequacy of the cDNA samples, a control PCR for bovine β-actin sequence was performed using a set of primers designed by Primer BLAST software (forward, 5’-GAGCGTGGCTACAGCTTCAC-3’; reverse, 5’-CATTGCCGATGGTGATGA-3’). The RT-PCR was carried out in a 25 μL reaction mixture containing 2.5 μL 10 X buffer, 2 mM MgCl_2_, 2.5 U of AmpliTaq Gold DNA Polymerase (Life Technologies, Monza, Italy), 480 nM of each primer and 200 mM of each dNTP and 50 ng DNA extract. The PCR program was 95°C for 3 min, followed by 35 cycles of denaturation at 95°C for 30 s, annealing at 60°C for 30 s and extension at 72°C for 1 min. A final extension step at 72°C for 7 min was performed in each PCR assay. Gel electrophoresis of 20 μL amplified DNA demonstrated a product of the expected size. In each experiment, a blank sample consisting of reaction mixture without DNA was included. As far as BPV E5 cDNA analysis a positive sample consisting of cloned BPV-2 (a kind gift by Dr. A. Venuti) and a BPV-13 positive sample (a kind gift by Dr A. A. Alfieri) were utilized. Amplified DNA was purified through silicagel membranes by using the QIAquick PCR quantification kit according to the manufacturer’s instructions (Qiagen, Milan, Italy). The RT-PCR products, after separation by gel electrophoresis, were sequenced using a Sanger method-based automatic sequencer (ABI PRISM 3100 Genetic Analyzer, Life Technologies, Monza, Italy). Furthermore, the amplified Mincle DNA from healthy and pathological bladders was cloned into pGEM-T vector using the pGEM-T Easy Vector System (Promega, Milan, Italy) and sequenced in the automated apparatus (ABI PRISM 3100 Genetic Analyzer, Life Technologies).

### Quantitative Real Time-PCR analysis (qRT-PCR) for Mincle

For qRT-PCR analysis, 500ng RNA were reverse-transcribed using the iScript cDNA Synthesis Kit (Bio-Rad Laboratories, Milan, Italy) and the reaction was incubated at 25°C for 5min, 42°C for 30min, 85°C for 5min, and then kept at 4°C for 5min. Real Time reactions were performed using SsoFast EvaGreen Supermix (Bio-Rad Laboratories, Milan, Italy). For the detection of Mincle specific primers (as described above) were used. All reactions were performed in triplicate and β-actin was used as the internal standard (forward primer 5’- TAGCACAGGCCTCTCGCCTTCG-3’; reverse primer 5’- GCACATGCCGGAGCCGTTGT-3’).

### Western blot analysis on bladder samples for mincle protein

Healthy and diseased bladders were solubilized for 2 h at 4°C in lysis buffer containing 50 mM Tris-HCl pH 7.5, 150 mM NaCl, 1% Triton X-100. Immediately prior to use, the following reagents were added: 1 mM DTT, 2 mM PMSF, 1.7 mg/ml Aprotinin, 25 mM NaF, 1 mM Na_3_VO_4_ (Sigma-Aldrich, Milan, Italy).

Lysates were clarified at 15000 rpm for 30 min. The protein concentration was measured using the Bradford assay (Bio-Rad Laboratories, Milan, Italy). For Western blotting, 100 μg of lysate proteins were heated at 100°C in 4X premixed Laemmli sample buffer (Bio-Rad Laboratories, Milan, Italy). Proteins were subjected to sodium dodecyl sulfate—polyacrylamide gel electrophoresis (SDS—PAGE) under reducing conditions.

After electrophoresis, proteins were transferred on nitrocellulose filter membranes (GE Healthcare Life Sciences, Chalfont St Giles, UK) for 1h at 10 V in 192 mM glycine/25 mM Tris-HCl (pH 7.5)/10% methanol using a Trans-Blot SD Semi Dry cell (Bio-Rad Laboratories, Milan, Italy) according to the manufacturer’s instructions. The membranes were blocked with 5% bovine serum albumin (BSA) in Tris-buffered saline (TBS, pH 7.5) for 1 h at room temperature, washed with TBS-0.1% Tween. Then, filters were probed with anti-Mincle (CLEC4E (P-15): sc-161486, (Santa Cruz Biotechnology, CA, USA) diluted 1:1000 in 5% BSA for an overnight incubation at 4°C. After three washes in Tris-buffered saline, membranes were incubated with horseradish peroxidase-conjugated anti-goat IgG (Santa Cruz Biotechnology, CA, USA) for 1 h at room temperature. After appropriate washing steps, bound antibody was visualized by an enhanced chemiluminescence system (Western Blotting Luminol Reagent, Santa Cruz Biotechnology, CA, USA). Images were acquired with Image Lab Software version 2.0.1 (Bio-Rad Laboratories, Milan, Italy).

### Immunohistochemistry

All pathological samples were immunostained and sections of normal bovine urinary bladder mucosa were tested in parallel as control using the avidin-biotin-peroxidase complex (ABC) method with the Vectastain ABC kit (Vector Laboratories, Inc., CA, USA). Briefly, paraffin sections were deparaffinized, and blocked for endogenous peroxidase in 0.3% H_2_O_2_ in methanol for 20 min. Antigen enhancement was performed by pretreating with microwave heating (twice for 5 min each at 750 W) in citrate buffer pH 6.0. The slides were washed three times with phosphate buffered saline (PBS; pH 7.4, 0.01 M), then incubated for 1 h at room temperature with normal rabbit serum (Vector Laboratories Inc., CA, USA) diluted at 20%. Polyclonal goat anti-CLEC 4E, anti-Fc ε RIγ, anti-Card9 and a monoclonal mouse anti-Syk (Santa Cruz Biotechnology Inc., CA, USA) primary antibodies diluted at 1 in 200, 1 in 400, 1 in 300 and 1 in 50, respectively, in phosphate buffered saline (PBS), were applied overnight at room temperature in a humid chamber. The slides were washed three times with PBS, then incubated for 30 min with appropriate biotinylated secondary antibody anti-goat (Vector Laboratories Inc., CA, USA) diluted at 1 in 200 in PBS. Sections were washed three times with PBS and then incubated with Vectastain ABC reagent (Vector Laboratories Inc., CA, USA) in a humid chamber at room temperature. Colour development was obtained by treatment with 3, 3’-diaminobenzidine (Vector Laboratories Inc., CA, USA) for 2–10 min. Sections were counterstained with Mayer’s haematoxylin. The primary antibody was omitted and replaced by antibody diluent in negative controls in which no background staining was detected.

## Results

**Fig 1:**
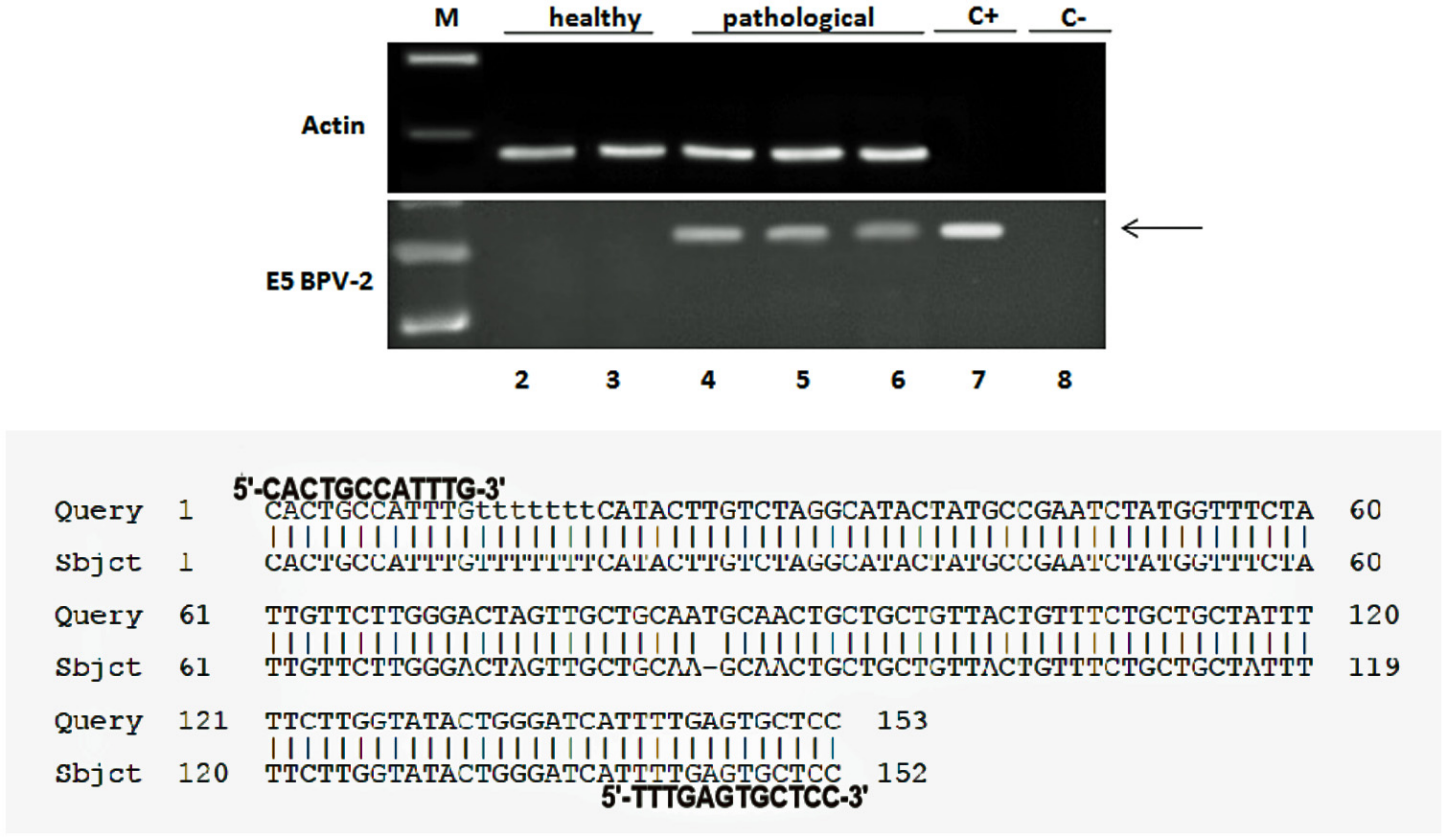
BPV 2 E5 oncoprotein and sequencing. Assessment of BPV-2 E5 cDNA by reverse transcriptase polymerase chain reaction (RT-PCR). M: 50 bp molecular marker (HyperLadder II Bioline); 2–3: healthy urinary bladder samples; 4–6: bladder tumor samples; 7: positive control (cloned BPV-2 DNA); 8: negative control (no DNA added). The arrow indicates the position of the 154 bp BPV-2 E5 PCR product.

**Fig 2:**
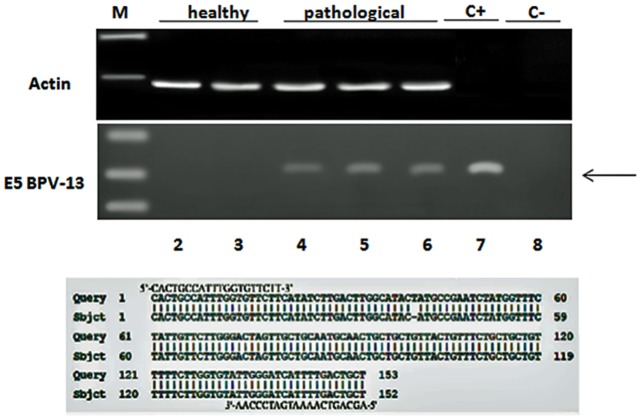
BPV 13 E5 oncoprotein and sequencing. Assessment of BPV-13 E5 cDNA by reverse transcriptase polymerase chain reaction (RT-PCR). M: 50 bp molecular marker (HyperLadder II Bioline); 2–3: healthy urinary bladder samples; 4–6: bladder tumor samples; 7: positive control (BPV-13 DNA); 8: negative control (no DNA added). The arrow indicates the position of the 153 bp BPV-13 E5 PCR product.

As abortive infections of BPV-2 and BPV-13 are commonly associated with urothelial tumors of the urinary bladder of cattle, firstly, we studied whether transcripts of E5 protein, the virus major oncoprotein, were present in the tumors. RT-PCR showed both BPV-2 and BPV-13 E5 transcripts in tumor cells only; no E5 transcripts were detected in healthy bladder samples. The presence of BPV-2 and BPV-13 E5 RNA was confirmed by sequencing of amplicons according to BPV-2 sequence M20219.1 and to BPV-13 sequence JQ798171.1 (Figs 1 and 2). Microscopic patterns of urothelial cancers and detection of papillomavirus E5 transcripts are summarized in Table 1.

**Table 1:** Histologic diagnosis of urothelial cancer and its association with Deltapapillomavirus.

Case Number	Microscopic patterns	BPV-2 E5 cDNA	BPV-13 E5 cDNA
1	HG Papillary urothelial carcinoma	+	+
2	HG Papillary urothelial carcinoma	+	+
3	HG Papillary urothelial carcinoma	+	-
4	LG Papillary urothelial carcinoma	+	-
5	LG Papillary urothelial carcinoma	NE	NE
6	Carcinoma in situ (CIS)	+	+
7	HG Invasive urothelial carcinoma	+	+
8	HG Invasive urothelial carcinoma	+	+
9	HG Invasive urothelial carcinoma	NE	NE
10	LG Invasive urothelial carcinoma	-	+

LG = Low-grade; HG = High-grade; + = presence of E5 cDNA; - = absence of E5 cDNA; NE = Not Examined.

**Fig 3:**
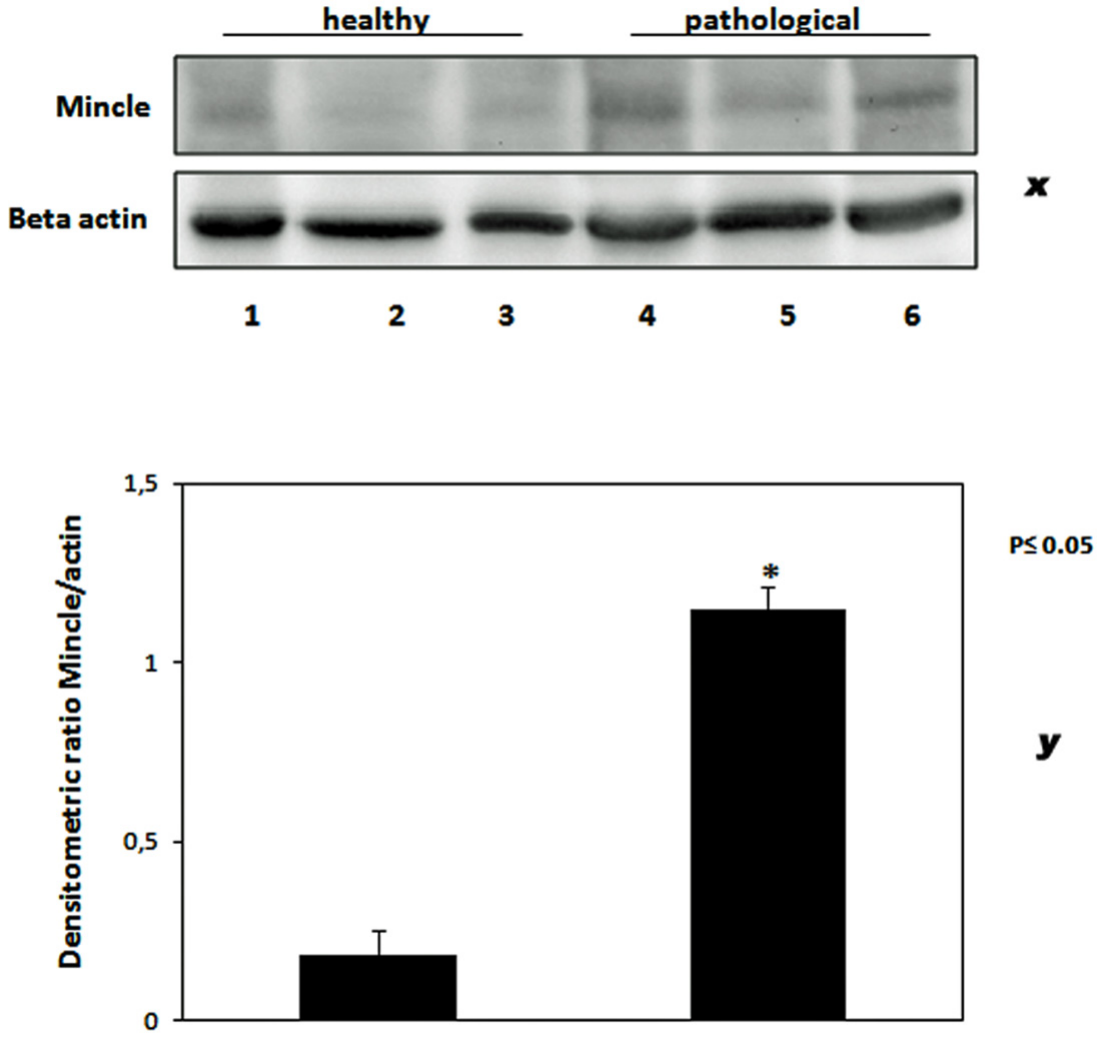
Mincle expression. (x) Western blot analysis showing a statistically significant overexpression of Mincle protein in tumor bladders. Lanes 1–3: urinary bladder from healthy animals. Lanes 4–6: neoplastic tissue from representative three animals with papillomavirus-associated tumors of the urinary bladder. Actin protein levels were detected to ensure equal protein loading. (y) Quantitative densitometric analysis of the filters was performed with Image Lab software (ChemiDoc; Bio-Rad Laboratories) and significance determined by the Student T-test (*, p≤0.05).

**Fig 4:**
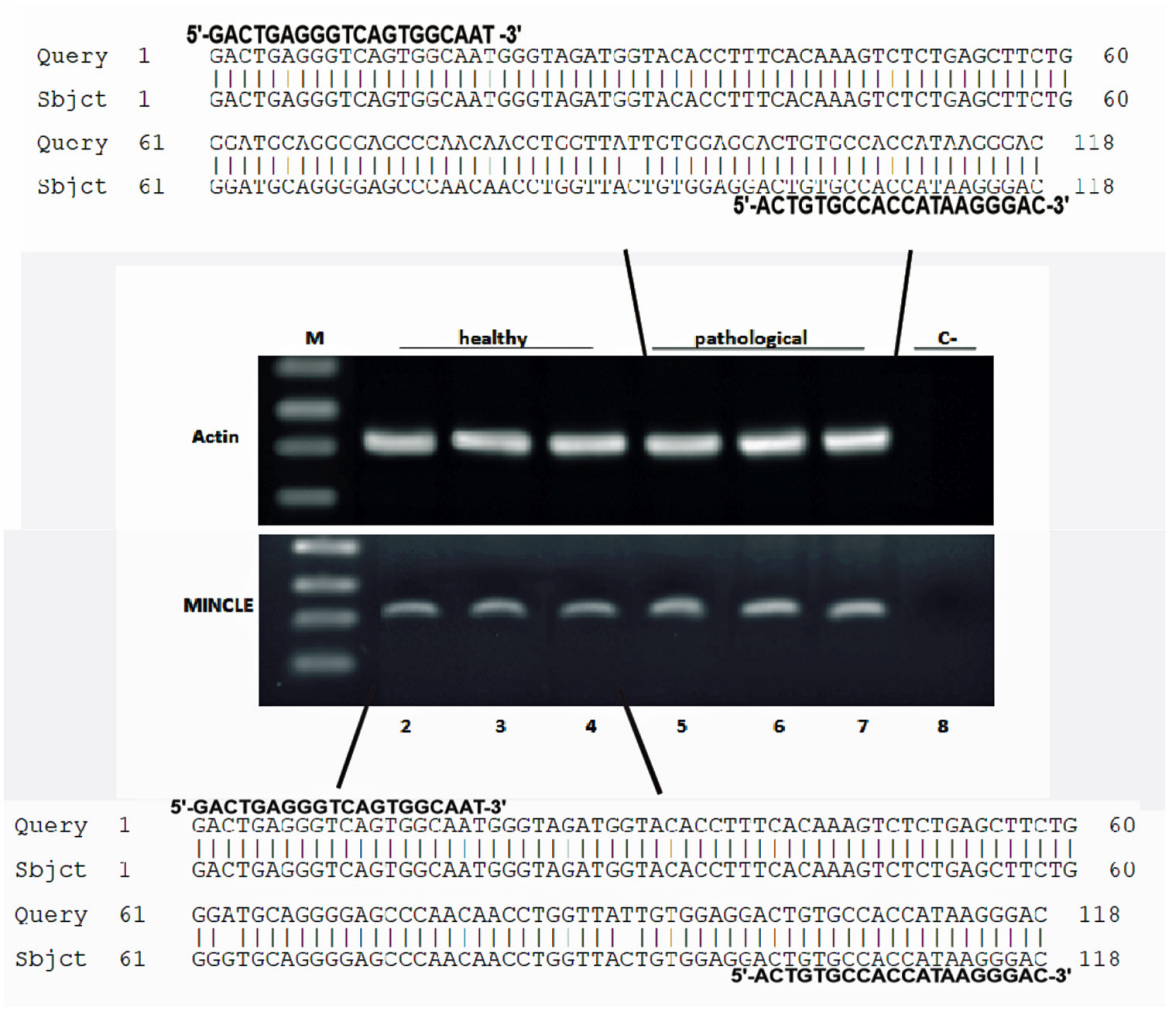
PCR amplification, cloning and sequencing of Mincle cDNA. Lane M, Molecular mass marker (1 kb DNA Ladder, Microtech). Lanes 2–4: Four normal (control) samples from healthy cattle. Lanes 5–7: nine representative tumor samples. Lane 8: negative control (no DNA added). The upper and lower parts of the figure show 99% and 98% homology, respectively, between the sequence of the amplicons, after cloning, and the sequence of bovine MINCLE deposited in GenBank (XM_010827920.1).

**Fig 5:**
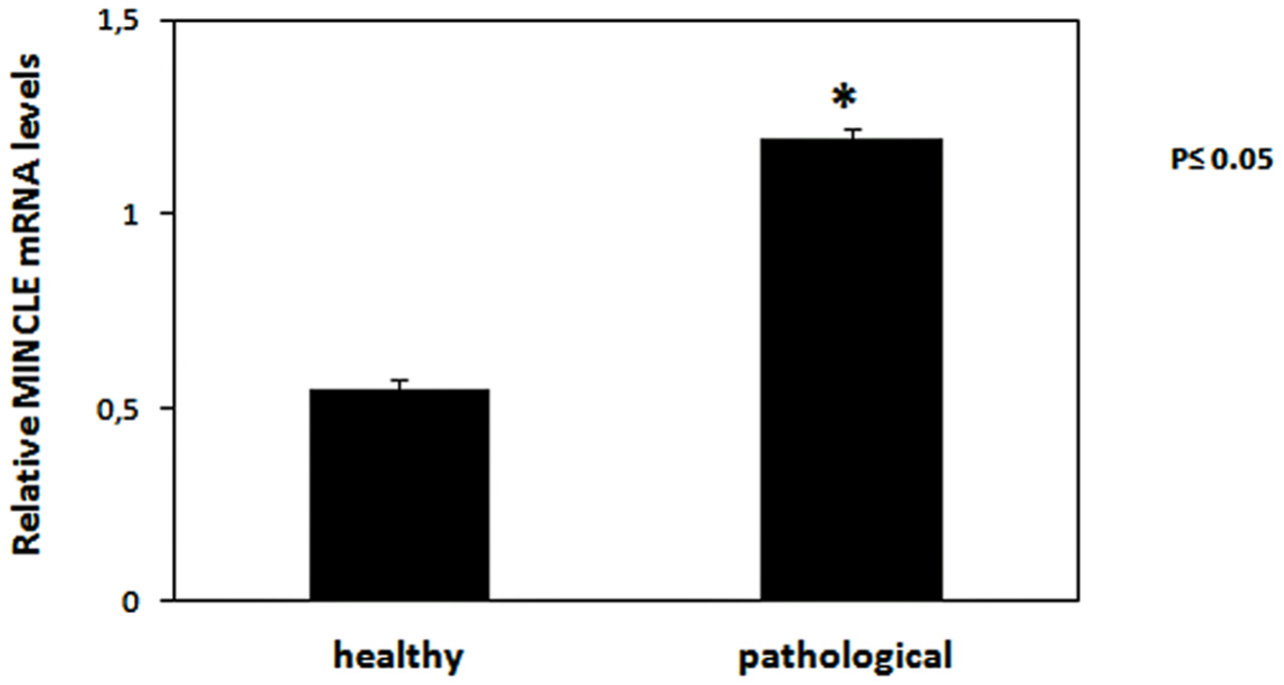
Quantitative real time PCR for MINCLE. The relative expression of MINCLE levels in neoplastic tissues. Relative mRNA levels, calculated using the ΔΔCT method, represent fold changes in comparison to bladder samples from healthy cattle. All values were normalized to the internal control β-actin. Results represent the means and standard deviations of three independent experiments performed in triplicate. (*, p≤0.05, vs urinary bladder from healthy animals).

Mincle protein was revealed both in healthy and pathological samples by western blot investigation; the protein appeared to be overexpressed in tumor samples as compared with healthy samples (Fig 3). RT-PCR revealed the presence of mincle transcripts both in normal and cancer samples. After cloning and sequencing, mincle transcripts showed a 98% and 99% identity, respectively, with Bos taurus mincle gene deposited in GenBank under accession number XM_010827920.1 (Fig 4). qRT-PCR detected a statistically significant increase of mincle mRNA levels in pathological samples (Fig 5).

**Fig 6:**
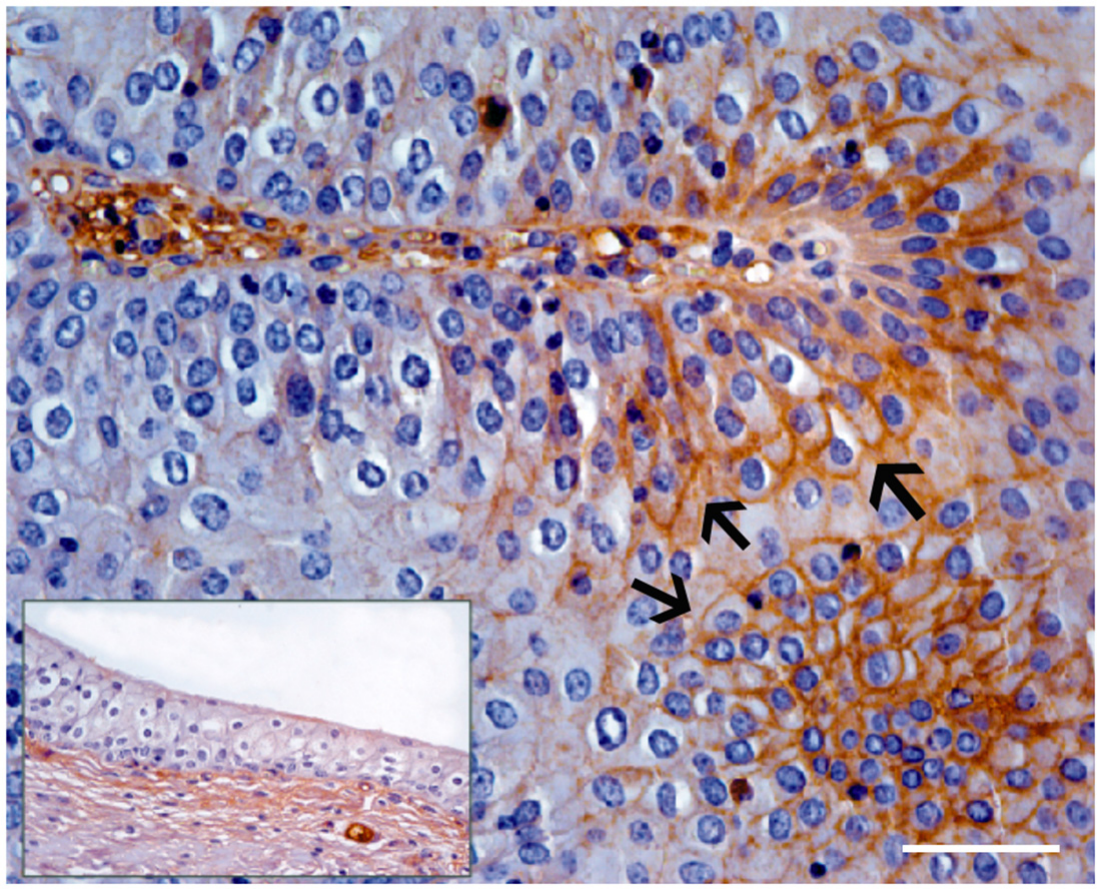
Papillary urothelial cancer. A membranous pattern of mincle expression was seen in endoluminal urothelial cell proliferation (arrows). Inset: Non immunoreactivity was seen in normal urothelial cells. Mag. 40X; Bar: 100 μm.

**Fig 7:**
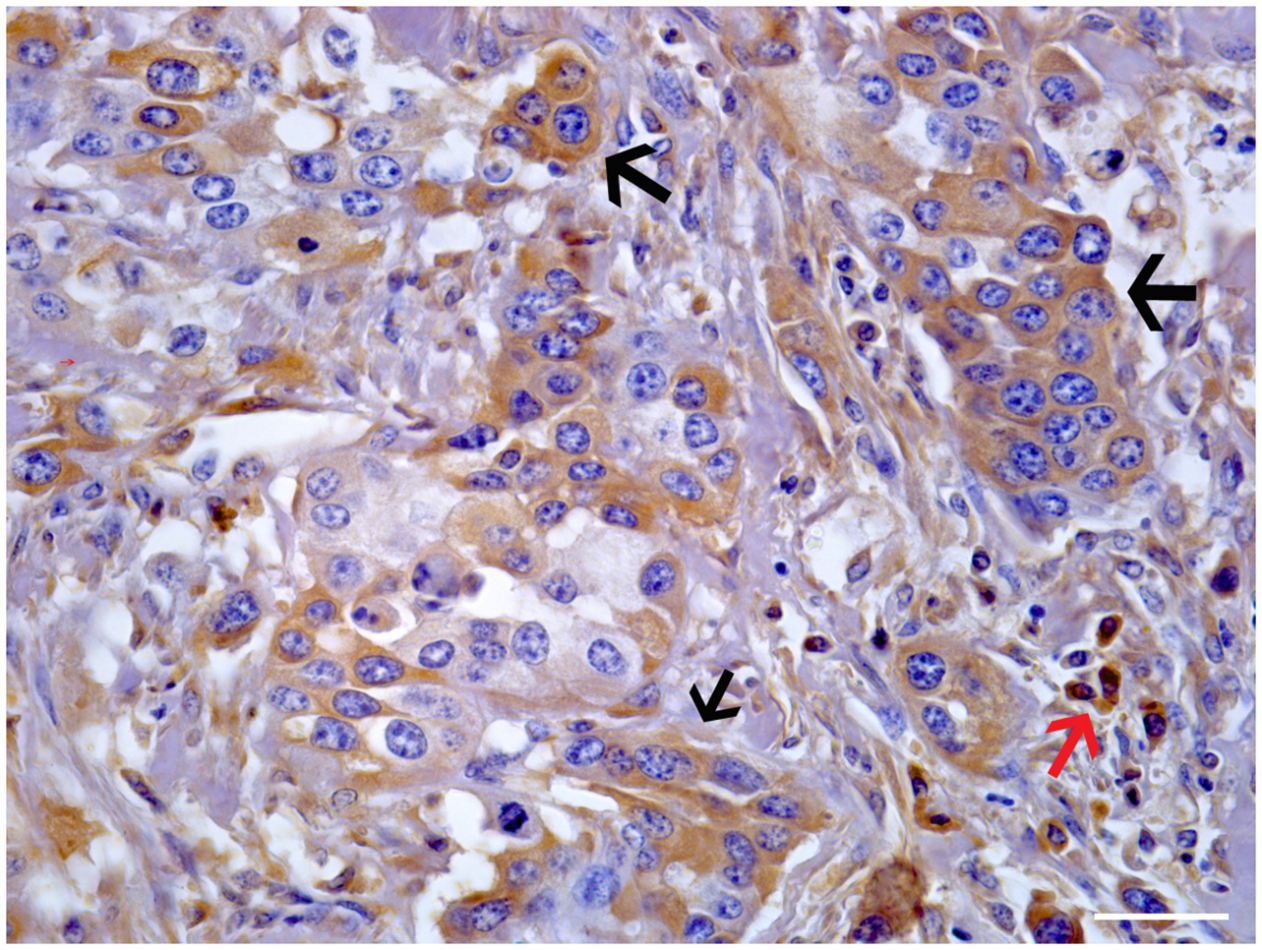
Invasive urothelial cancer. Clustered of neoplastic cells showed a strong cytoplasmic immunoreactivity for mincle (black arrows). Isolated immunoreactive neoplastic cells were also seen scattered in bladder wall. Immunoreactivity was also evident in tumor-associated macrophages (TAMs) (red arrow). Mag. 40X. Bar: 100 μm.

Morphologically, mincle protein was detected by immunohistochemical procedures in urothelial tumor cells only and not in normal urothelial cells. A membranous pattern of mincle immunoreactivity was seen in neoplastic cells of both low- and high-grade papillary urothelial tumors (Fig 6); in addition, scattered and clustered neoplastic urothelial cells of both low- and high-grade invasive urothelial cancer showed a strong immunoreactivity for mincle, prevalently in a cytoplasmic pattern appearance. Mincle expression was also detected in inflammatory cells scattered among cancer cells; in particular, receptor expression was seen in tumor-associated macrophages (TAM) (Fig 7).

**Fig 8:**
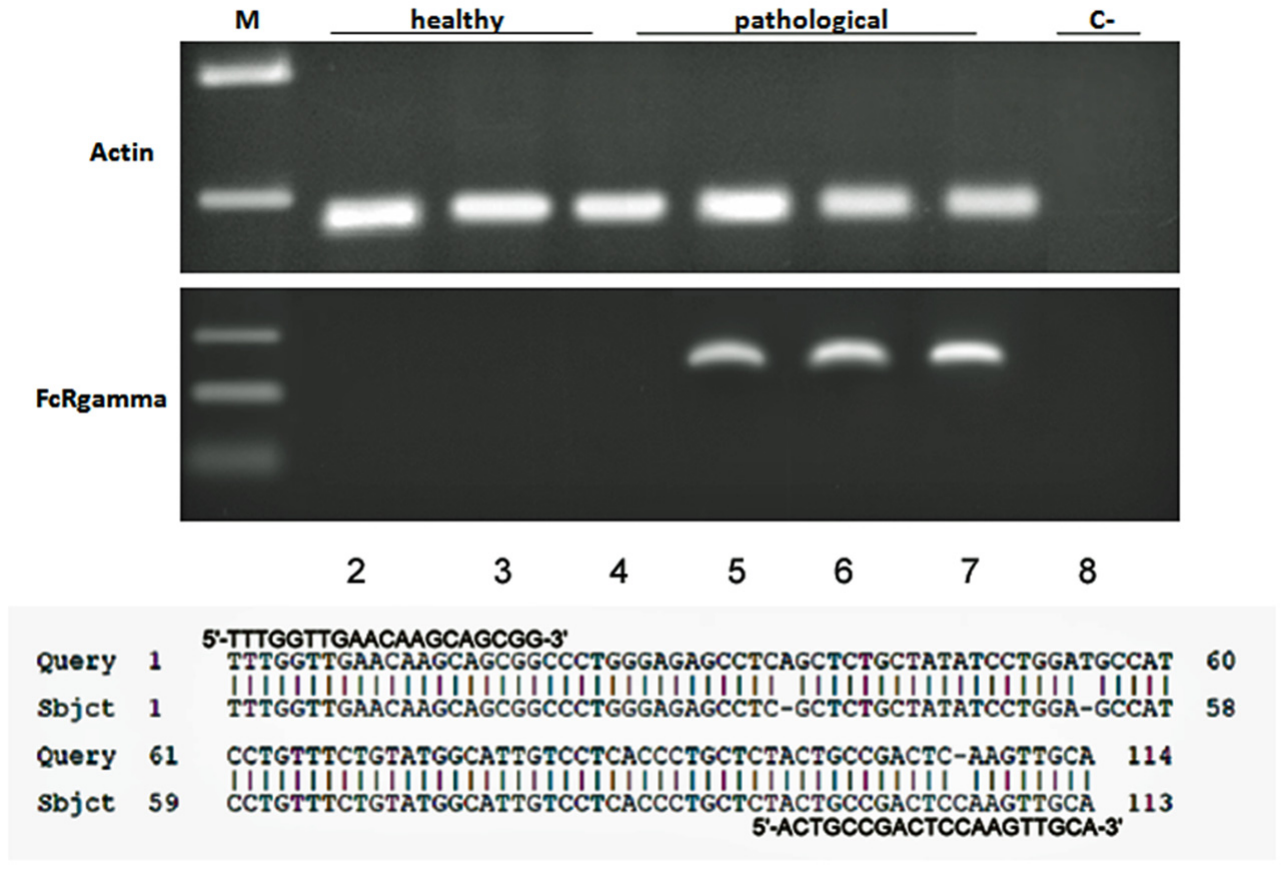
FcγR expression and sequencing. Assessment of FcγR mRNA expression by reverse transcriptase polymerase chain reaction (RT-PCR). M: molecular marker (HyperLadder II Bioline); 2–4: healthy urinary bladder samples; 5–7: bladder tumor samples; 8: negative control (no DNA added). The lower part of the figure shows an identity of 97% with Bos taurus FcγR1 (GenBank accession number: AF316499.1).

**Fig 9:**
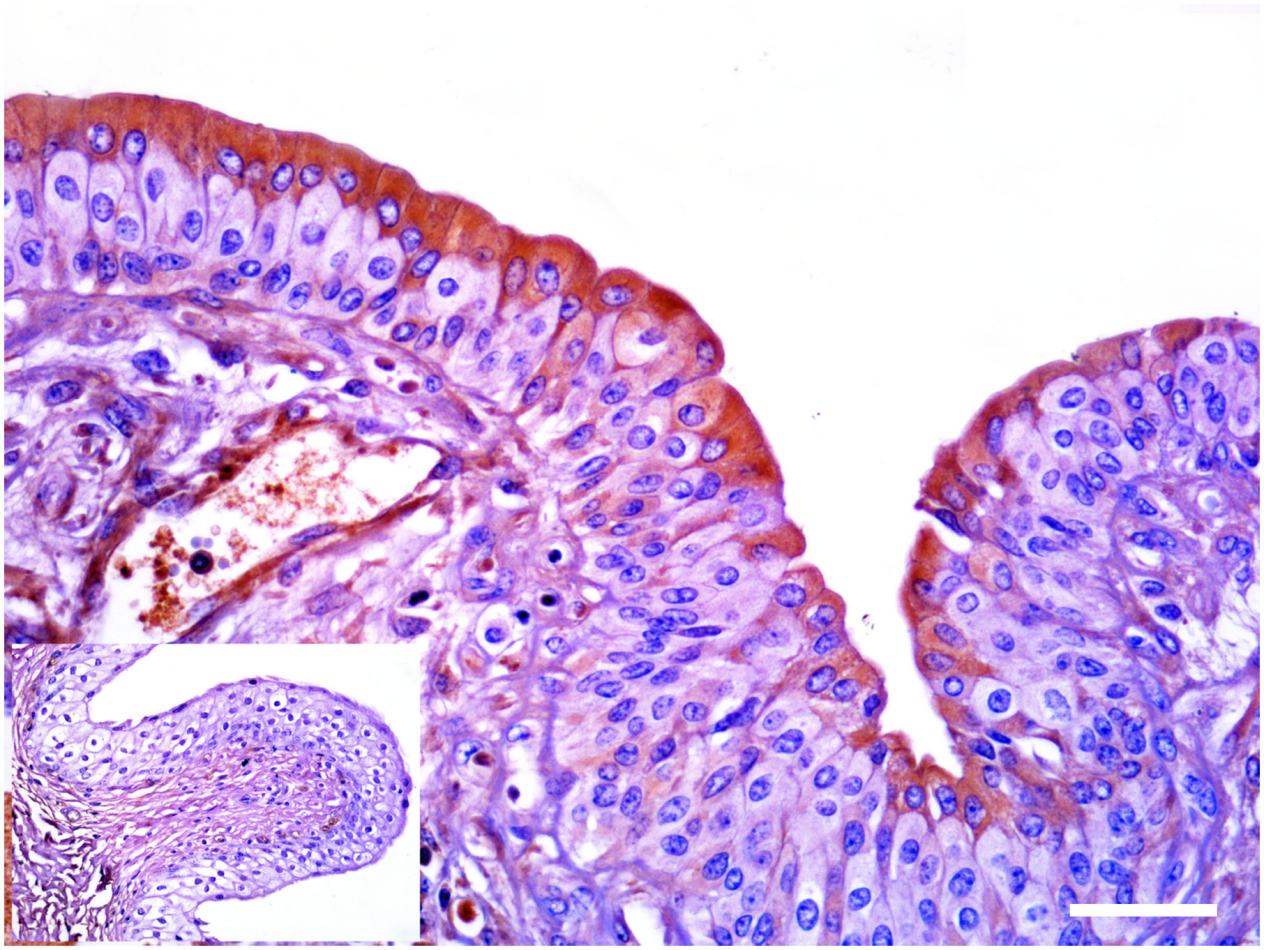
Morphological expression of FcγR in cancer cells. A strong cytoplasmic and membranous immunoreactivity showing an aberrant expression of FcγR was seen in urothelial cancer cells. Inset: No FcγR expression was detected in normal urothelial cells. Mag. 40X; Bar: 100 μm.

**Fig 10:**
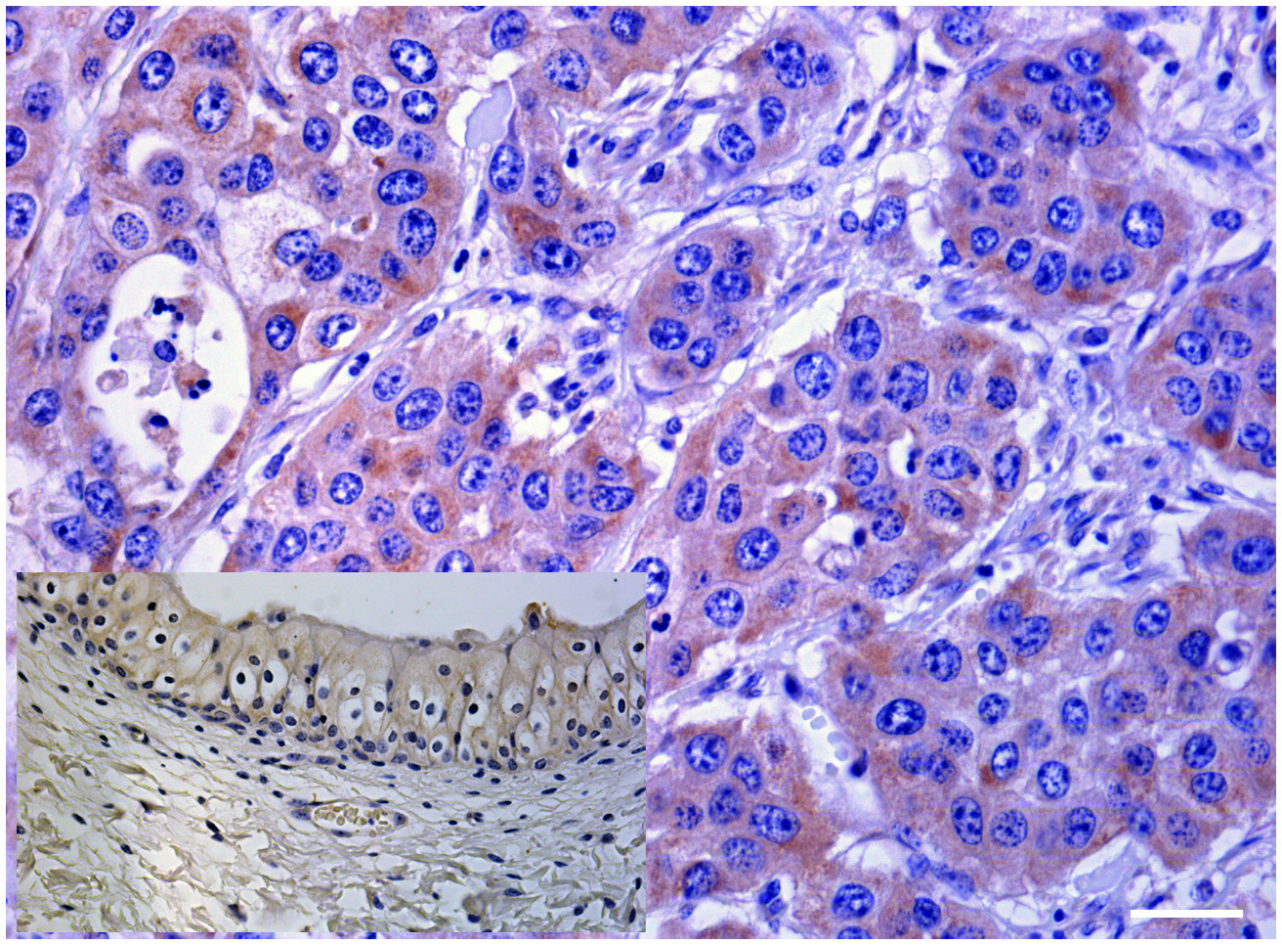
Morphological expression of Syk protein in cancer cells. A strong immunoreactivity showing an evident expression of Syk was seen in urothelial cancer cells. Inset: No immunoreactivity for Syk was detected in normal urothelial cells. Mag. 40X; Bar: 100 μm.

**Fig 11:**
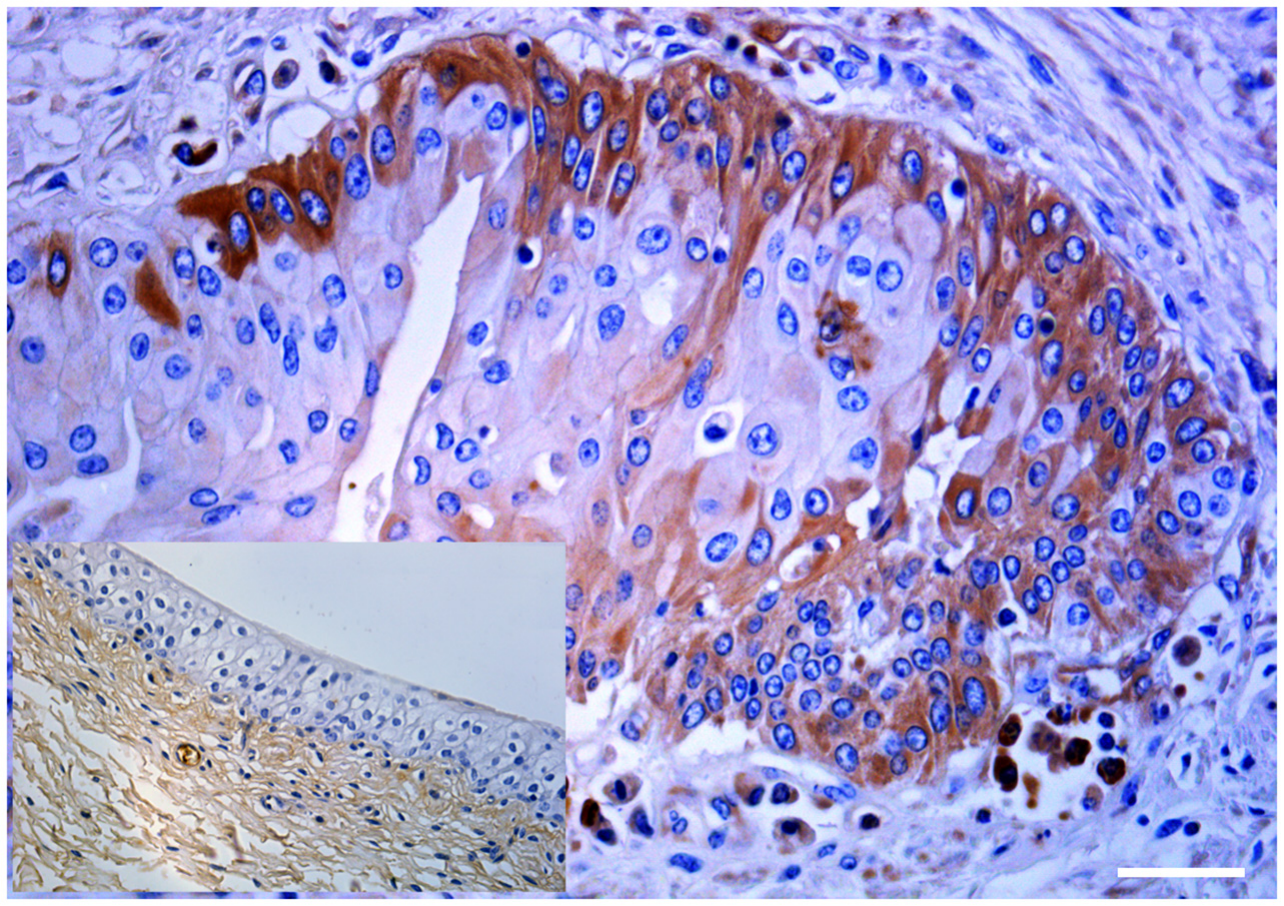
Morphological expression of Card9 in cancer cells. A strong cytoplasmic immunoreactivity showing a clear expression of Card9 was seen in urothelial cancer cells. Inset: No immunoreactivity for Card9 was seen in normal urothelial cells. Mag. 40X; Bar: 100 μm.

It is known that Mincle associates with the adaptor protein FcγR which is required for initiation of signaling by binding Syk which, in turn, activates a signaling cascade through Card9 and this event leads to the induction of cytokine and chemokine such as CXCL2 [5, 6]. Therefore, we investigated FcγR mRNA expression both in normal and pathological bladder samples by RT-PCR. Amplicons, sequencing of which revealed to be FcγR transcripts showing an identity of 97% with Bos taurus FcγRI (GenBank accession number: AF316499.1), were detected in cancer samples only (Fig 8). Morphologically, a strong cytoplasmic and membranous immunoreactivity revealing an unexpected, aberrant expression of FcγRI was evident in urothelial cancer cells (Fig 9). Then, we investigated whether a morphological expression of the Syk-Card9 axis could be detected in urothelial cells of healthy and neoplastic samples. A strong immunoreactivity revealing an evident Syk expression was clearly detected in urothelial cancer cells only (Fig 10). Furthermore, also Card9 protein expression was immunohistochemically seen in urothelial tumor cells. No immunoreactivity for Card9 was manifest in urothelial cells of healthy samples (Fig 11).

**Fig 12:**
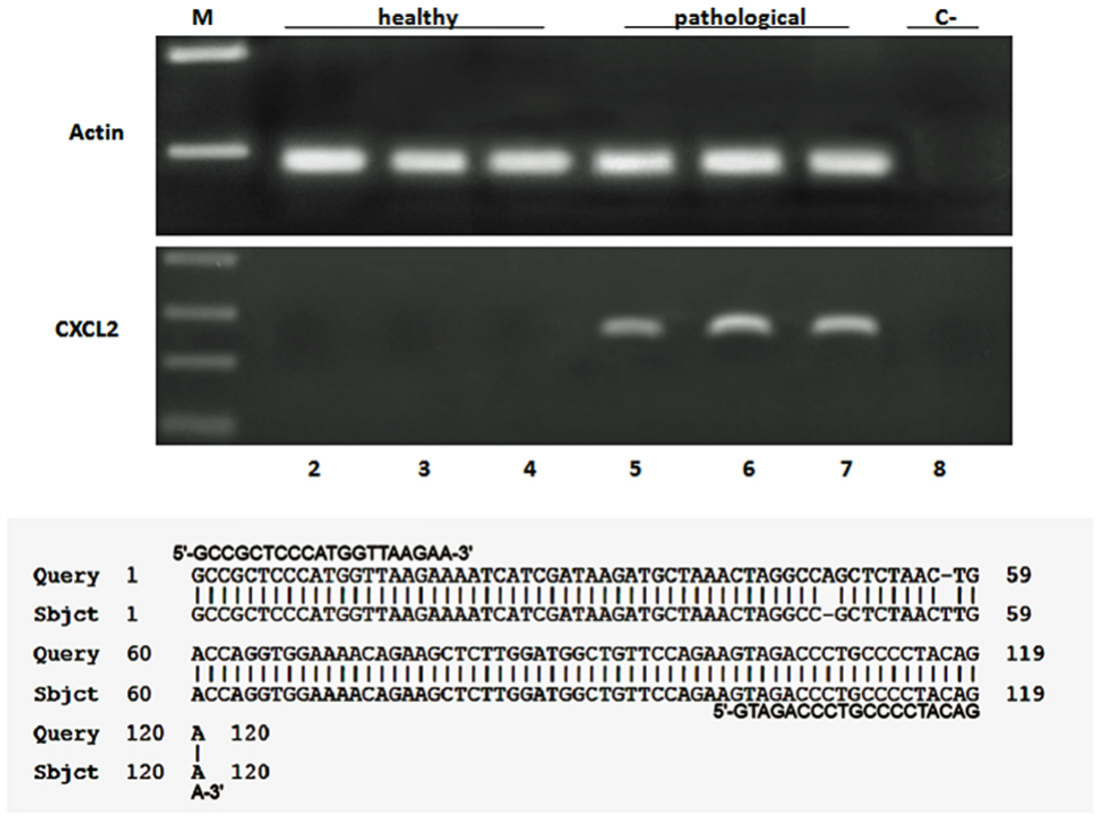
CXCL2 expression and sequencing. Assessment of CXCL2 mnRNA expression by reverse transcriptase polymerase chain reaction (RT-PCR). M: molecular marker (HyperLadder II Bioline); 2–4: healthy urinary bladder samples; 5–7: bladder tumor samples; 8: negative control (no DNA added). The lower part of the figure shows a 98% identity with Bos taurus CXCL2 (GenBank accession number: NM_1742993).

Finally, we studied mRNA expression of CXCL2, a pro-inflammatory chemokine. RT-PCR revealed a presence of cDNA in cancer samples only; sequences obtained from the amplicons confirmed to be CXCL2 transcripts which showed a 98% identity with Bos taurus CXCL2 (GenBank accession number: NM_174299.3) (Fig 12).

## Discussion

We detected mincle expression in papillomavirus-associated urothelial tumors of the urinary bladder in cattle. This is the first report of the expression of mincle in veterinary oncology and the first observation describing the expression of functional mincle receptor in neoplastic cells in medical literature. Morphologically, mincle protein was seen in urothelial neoplastic cells only but not in normal urothelial cells, which might mean that level of mincle expression is so low in steady-state condition as not to be detected by immunohistochemical procedures. We do not exclude that mincle detected in healthy bladder comes from clusters of lymphoid and macrophage cells that usually are present in bladder wall of healthy cows.

It is possible that genomic alterations leading to dysregulation of some CLR genes may be responsible for mincle as well as FcγRI expression in urothelial tumor cells; it is also conceivable that mincle expression could be activated by some endogenous ligands because numerous necrotic foci, that can cause the release of self-components are seen in urothelial tumors of the urinary bladder of cattle. Therefore, mincle may be involved in the induction of a pro-inflammatory response after sensing components released from dead cells. According to Suzuki et al. [13], many endogenous ligands for mincle receptor are still to be identified.

It has been suggested that urothelial cells (including bladder cancer cells themselves) are able to produce cytokines and chemokine and are involved in the presentation of cancer cell antigens to cells of the immune system [27]. Indeed, some chemokine receptors have been detected on urothelial cancer cells [28]. Recently, some ligands for chemokine receptors CXCR4 and CXCR7, have been found to be secreted by urothelial cancer of the human urinary bladder [29]. Therefore, it has been suggested that urothelial cancer cells have the ability to function as antigen-presenting cells (APCs). This ability seems to be clinically relevant as it would influence recurrence of bladder cancer [30].

Although mincle expression in urothelial tumor cells warrants further study to better understand the role of this receptor in bladder cancer, this report has exciting clinical implications in comparative medicine. Bacillus Calmette-Guérin (BCG) immunotherapy is currently the most effective treatment of non-muscle invasive bladder cancer in man [31]. Although BCG has been used for the treatment of bladder cancer for nearly 40 years, the way in which it achieves its therapeutic effect remains a matter of investigation [27]. It has been shown that bladder cancer cell lines are able to internalize BCG [32]. It has been hypothesized that internalization of BCG by bladder cancer cells is governed by the presence of certain unknown oncogenic aberrations that activate macropinocytosis [27]. Therefore, bladder tumor cells might have a role in the initial recognition and processing of BCG, leading to immune system recruitment; however, pathogenetic mechanism(s) must be elucidated [27]. Toll-like receptors have been found on cultured normal and urothelial tumor cells and might have a role in antitumor immunity of nonmuscle invasive tumors [33].

Many questions remain still unanswered and many areas require further investigation as studies have not yet evaluated the role of internalization of BCG by bladder cancer cells *in vivo* [27]. Our study allows us to hypothesize that functional mincle receptor of neoplastic urothelial cells may have a crucial role in recognizing and internalizing BCG. Our suggestion appears to be corroborated by recent reports showing that expression of mincle contributes to the control of *Mycobacterium bovis* BCG infection [34].

Future studies will hopefully be very useful to provide insights in the role of mincle receptor of urothelial cancer cells in antitumor immunotherapy. In this context, mincle receptor can be investigated in depth in naturally occurring bovine urothelial tumors and cattle may serve as a natural animal model. It has been shown that there is a high degree of sequence identity between the human and bovine form of mincle and they interact with ligands in a very similar way [35].

## Supporting Information

S1 Fig S1 sequencing after cloning of Mincle from heathy samples. (PDF)

S2 Fig S2 sequencing after cloning of Mincle from pathological samples. (PDF)
