# Supplementary material for: Mincle, an Innate Immune Receptor, Is Expressed in Urothelial Cancer Cells of Papillomavirus-Associated Urothelial Tumors of Cattle
Source: PLoS One. 2015 Oct 29;10(10):e0141624. doi: 10.1371/journal.pone.0141624 (PMC4626233; doi:10.1371/journal.pone.0141624)
Supplement: S1 Fig — (PDF) [file pone.0141624.s001.pdf]

BLAST ®

Basic Local Alignment Search Tool

NCBI/ BLAST/ blastn suite-2sequences/ **Formatting Results - VH1HBTZ011N**

Formatting options
Download
Blast report description

Blast 2 sequences

Nucleotide Sequence (118 letters)

RID VH1HBTZ011N (Expires on 07-30 15:52 pm)

**Query ID**	lcl|Query_252699
**Description**	None
**Molecule type**	nucleic acid
**Query Length**	118

**Subject ID**	lcl|Query_252701
**Description**	None
	See details
**Molecule type**	nucleic acid
**Subject Length**	118
**Program**	BLASTN 2.2.32+

Graphic Summary

Distribution of 1 Blast Hits on the Query Sequence

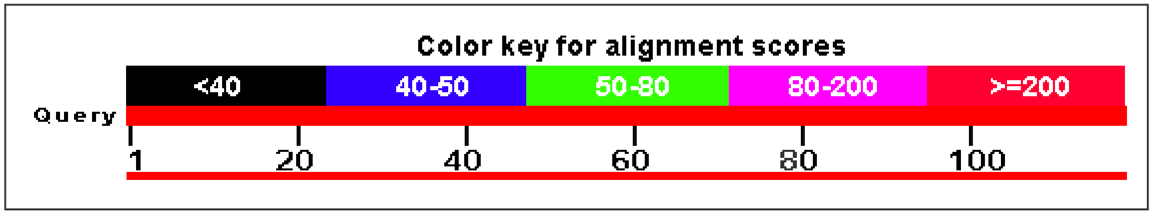

Dot Matrix View

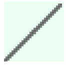

Descriptions

Sequences producing significant alignments:

Description	Max score	Total score	Query cover	E value	Ident	Accession
None provided	207	207	100%	3e-59	98%	Query_252701

Alignments

Sequence ID: lcl|Query_252701 Length: 118 Number of Matches: 1
Range 1: 1 to 118

Score	Expect	Identities	Gaps	Strand	Frame
207 bits(112)	3e-59()	116/118(98%)	0/118(0%)	Plus/Plus	

Features:

```
Query  1     GACTGAGGGTCAGTGGCAATGGGTAGATGGTACACCTTTCACAAAGTCTCTGAGCTTCTG  60
             ||||||||||||||||||||||||||||||||||||||||||||||||||||||||||||
Sbjct  1     GACTGAGGGTCAGTGGCAATGGGTAGATGGTACACCTTTCACAAAGTCTCTGAGCTTCTG  60

Query  61    GGATGCAGGGGAGCCAACAACCTGGTTATTGTGGAGGACTGTGCCACCATAAGGAC  118
             || ||||||||||||||||||||||||| |||||||||||||||||||||||||||||
Sbjct  61    GGGTGCAGGGGAGCCCAACAACCTGGTTACTGTGGAGGACTGTGCCACCATAAGGAC  118
```
