# Supplementary material for: Mincle, an Innate Immune Receptor, Is Expressed in Urothelial Cancer Cells of Papillomavirus-Associated Urothelial Tumors of Cattle
Source: PLoS One. 2015 Oct 29;10(10):e0141624. doi: 10.1371/journal.pone.0141624 (PMC4626233; doi:10.1371/journal.pone.0141624)
Supplement: S2 Fig — (PDF) [file pone.0141624.s002.pdf]

BLAST ®

Basic Local Alignment Search Tool

NCBI/ BLAST/ blastn suite-2sequences/ **Formatting Results - VH1N3TXU11N**
Formatting options
Download
Blast report description

Blast 2 sequences

Nucleotide Sequence (118 letters)

RID VH1N3TXU11N (Expires on 07-30 15:54 pm)

**Query ID**	lcl|Query_19241	**Subject ID**	lcl|Query_19243
**Description**	None	**Description**	None<br>See details
**Molecule type**	nucleic acid	**Molecule type**	nucleic acid
**Query Length**	118	**Subject Length**	118
		**Program**	BLASTN 2.2.32+

Graphic Summary

Distribution of 1 Blast Hits on the Query Sequence

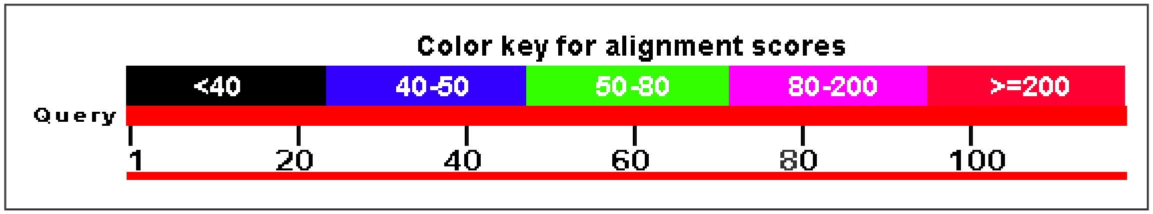

Dot Matrix View

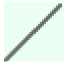

Descriptions

Sequences producing significant alignments:

Description	Max score	Total score	Query cover	E value	Ident	Accession
None provided	213	213	100%	7e-61	99%	Query_19243

Alignments

Sequence ID: lcl|Query_19243 Length: 118 Number of Matches: 1
Range 1: 1 to 118

Score	Expect	Identities	Gaps	Strand	Frame
213 bits(115)	7e-61()	117/118(99%)	0/118(0%)	Plus/Plus	

Features:

```
Query  1    GACTGAGGGTCAGTGGCAATGGGTAGATGGTACACCTTTCACAAAGTCTCTGAGCTTCTG  60
            ||||||||||||||||||||||||||||||||||||||||||||||||||||||||||||
Sbjct  1    GACTGAGGGTCAGTGGCAATGGGTAGATGGTACACCTTTCACAAAGTCTCTGAGCTTCTG  60

Query  61   GGATGCAGGGGAGCCCAACAACCTGGTTATTGTGGAGGACTGTGCCACCATAAGGGAC  118
            ||||||||||||||||||||||||||||| ||||||||||||||||||||||||||||
Sbjct  61   GGATGCAGGGGAGCCCAACAACCTGGTTACTGTGGAGGACTGTGCCACCATAAGGGAC  118
```
